# Can we replace curation with information extraction software?

**DOI:** 10.1093/database/baw150

**Published:** 2016-12-26

**Authors:** Peter D. Karp

**Affiliations:** Bioinformatics Research Group, SRI, International, 333 Ravenswood Ave, Menlo Park, CA 94025, USA. Tel:650-859-4358; Fax: 650-859-3735; E-mail: pkarp@ai.sri.com

## Abstract

Can we use programs for automated or semi-automated information extraction from scientific texts as practical alternatives to professional curation? I show that error rates of current information extraction programs are too high to replace professional curation today. Furthermore, current IEP programs extract single narrow slivers of information, such as individual protein interactions; they cannot extract the large breadth of information extracted by professional curators for databases such as EcoCyc. They also cannot arbitrate among conflicting statements in the literature as curators can. Therefore, funding agencies should not hobble the curation efforts of existing databases on the assumption that a problem that has stymied Artificial Intelligence researchers for more than 60 years will be solved tomorrow. Semi-automated extraction techniques appear to have significantly more potential based on a review of recent tools that enhance curator productivity. But a full cost-benefit analysis for these tools is lacking. Without such analysis it is possible to expend significant effort developing information-extraction tools that automate small parts of the overall curation workflow without achieving a significant decrease in curation costs.

Database URL:

## Introduction

Bourne et al. recently proposed (1) that to improve efficiency and decrease costs, biomedical databases must explore new business models and methodologies. They suggest three alternatives to traditional literature-based curation by professional curators that they presumably believe will decrease the costs of curation: ‘complete and accurate automated or semi-automated extraction of literature’, crowd sourcing of curation, and curation by authors of publications.

Although the costs of professional curation are surprisingly low (2) (on average the cost of curating one article for the EcoCyc database is roughly 10% of the open-access publication fee for publishing a biomedical article), here we consider the first alternative to professional curation. What progress has been made, what challenges remain, and how practical an alternative is automated or semi-automated information extraction? We will consider the other alternatives in a future perspective.

## Text mining as an alternative to professional curation

Extracting Information from written texts is a form of the natural-language understanding problem, an Artificial Intelligence problem that has remained unsolved for 60+ years. Although significant progress has been made in this field, information-extraction programs (IEPs) are not accurate or comprehensive enough to replace manual curation. One of the simpler IEP tasks involves recognizing the names of entities in biomedical texts, which is called the named-entity recognition problem. Error rates (computed as 1−*F*-score) for six state-of-the-art named-entity recognition tools for recognizing the names of genes, diseases, organisms, chemicals, and mutations in text (one object type per program) range from 6 to 46% (mean is 18%) (3). Other recent results on named-entity recognition come from the BioCreative V competition, involving recognition of chemical names and disease names; Table 2 of (4) lists results from 16 teams where the error rates range from 13 to 48% (mean is 24%). Recognizing named entities in biomedical texts is the first step in extracting more complex relationships among those entities. Ananiadou et al. (5) review a number of IEPs for extracting a wide range of types of bio-events from texts, where a bio-event is ‘a textual event specialised for the biomedical domain, normally a “dynamic” bio-relation in which at least one of the biological entities in the relationship is affected’. Table 1of (5) lists the error rates of 15 IEP programs, which range from 23 to 58% (mean is 45%). BioCreative V error rates for IEPs from 18 teams for extraction of chemical-induced disease relations range from 43 to 68% (mean is 57%) [Table 2 of (4)].

In contrast, a recent study of ours has shown the accuracy of manual curation to be very high (1.4% error rate for EcoCyc, 1.8% error rate for Candida Genome Database) (6). Thus, the 33 IEPs for recognizing *single* relations surveyed by (4, 5) have error rates that are 14–42 *times higher* than the error rates of manual curation.

But furthermore, note that to date, all IEP programs extract single narrow slivers of information, such as individual protein interactions. In contrast, curators for our EcoCyc database (7) gather an extremely broad set of data on gene function, enzyme activities, metabolic pathways, and regulatory networks, that is stored in 360 distinct database fields. No IEP program can extract anywhere near this breadth of information.

Some biomedical databases (e.g. Genbank) make no attempt to synthesize the literature or to arbitrate among conflicting statements because many such databases seek to mirror the structure of the literature: one database entry reflects the findings in one publication. But in review-level databases such as EcoCyc, one database entry corresponds to one biological entity, and curators seek to integrate many published findings about that entity. For example, EcoCyc curators synthesize multi-paragraph mini-review summaries for protein and pathway pages; they follow changes in the names of genes, proteins, and metabolites; and they summarize and resolve disagreements and conflicts in the literature—capabilities that far exceed what IEPs or other Artificial Intelligence techniques can do.

To generalize, the difficulty of automating curation (or, for that matter, of crowd-sourcing curation), will depend on the complexity of that curation. Different databases employ curation processes of varying complexity depending on the number of types of data they extract, the number of database fields that are populated by the curation effort, the amount of meta-data extracted (e.g. is extracted information annotated with evidence codes?), the amount of knowledge integration (interpretation and synthesis) that the curators perform, whether curators author mini-reviews, and the end uses to which the data will be put (curation of knowledge to form an executable metabolic model will be more difficult than curation of knowledge to create a web page that will be read by scientists).

## Semi-automated extraction as an alternative to professional curation

In my opinion, there is much more near-term potential for semi-automated text-mining approaches to accelerate curation work. But to date, results have been very limited. One success story is software developed by WormBase to perform article triage—categorizing the type of information contained in articles for assignment to an appropriate member of the curation staff (8). WormBase also developed software that identifies sentences within publications that contain words likely to be stating the cellular compartments in which proteins are localized, analyzes those sentences, and pre-fills a curation form that could then be approved or modified by a curator (9). An evaluation found the tool to be moderately accurate (*F*-score of .509 for dictyBase and .547 for TAIR). The tool was found to increase curator efficiency 2.5-fold for dictyBase and 10-fold for TAIR; an earlier study by these authors found that the time to curate cellular compartment information could be decreased by a factor of 8–15 (10).

At first glance these results seem quite significant, but the accuracy of these tools is limited, and a full cost-benefit analysis for these tools is lacking. As a database Principal Investigator, to decide whether to adopt a given new semi-automated extraction tool in the EcoCyc curation workflow, I want to see a clear cost/benefit analysis that will let me calculate the pay-back time for introducing a new semi-automated tool.

On the benefit side, I want to know how much total curator time the tool saves in a realistic curation workflow. For example, the earlier statement that the tool increased curator efficiency 2.5-fold for dictyBase almost certainly is a local analysis of the isolated task of curating cellular-compartment information only; certainly dictyBase curates many other types of information that were not affected by that 2.5-fold speedup. In a local analysis of cellular-compartment curation the curator can ignore all parts of a article except for the cellular-compartment information, but in a real-world curation setting the curators probably still have to read most of the publication to extract other facts sought by the database that might be present in the article—it is difficult to be sure all facts within an article have been found without reading most of the article. Curation time savings would also be insignificant if the fraction of curated articles containing cellular-compartment facts was a small fraction of all curated articles. That is, if we increase curation speed by a factor of 2.5 for 5% of curated articles (imagine that only 5% of articles contain cellular location information), total time saved will be small. These aspects were not considered by (9, 10). One way to measure the real benefit is to measure total curation throughput before and after the introduction of the new tool. A second approach would is to estimate the change in throughput by multiplying the increase in efficiency in a specific curation task (e.g. the speed factor of 2.5) times the fraction of total curator time spent on that curation task (e.g. the 5% of curated articles).

On the cost side, I would want to know the cost of adding the software tool into a curation workflow, and the cost of maintaining that tool (e.g. upgrades and bug fixes). Imagine that it takes one month of programmer time to introduce a publication-triage program into the EcoCyc curation workflow. In EcoCyc we spend only four hours per month manually triaging publications, so it would take 3 years for us to recoup the programmer cost—assuming the program eliminated all curator triage time. Yet those four hours per month amount to 0.83% of our curator time—an insignificant savings. If we are going to make a significant dent in curation costs, we must try to optimize those curation tasks that take a significant fraction of curation resources.

## Conclusions

By all means, let us find ways to improve upon the limited productivity boosts achieved by semi-automated extraction systems to date. But authors must provide clear cost-benefit calculations for such tools in the context of realistic curation workflows so that we can predict likely cost savings to curation projects. In addition we need clear metrics for the accuracy of both manual curation and IEPs that demonstrate that IEPs can achieve the high quality that manual curators can (based on the few studies available of manual-curation accuracy).

Currently, fully automated information extraction will be practical only for those databases that extract narrow ranges of information, and whose end users can tolerate high error rates. These constraints mean that for the vast majority of curated biomedical databases, automated information extraction is currently not a practical alternative to professional curation, nor will it be so in the near term. Therefore, funding agencies should not hobble the curation efforts of existing databases out of wishful thinking that a problem that has stymied Artificial Intelligence researchers for >60 years will be solved tomorrow.

## References

[1] Bourne P.E. Lorsch J.R. Green E.D. (2015) Sustaining the big-data ecosystem. Nature, 527, S16–S17. 26536219 10.1038/527S16a

[2] Karp P.D. How much does curation cost? submitted for publication.

[3] Wei C.H. Kao H.Y. Lu Z. (2013) PubTator: a web-based text mining tool for assisting biocuration. Nucleic Acids Res., 41(Web Server issue), W518–W522. 23703206 10.1093/nar/gkt441 PMC3692066

[4] Wei C.H. Peng Y. Leaman R. Overview of the biocreative v chemical-disease relation (cdr) task. In Proceedings of the Fifth BioCreative Challenge Evaluation Workshop, pp. 1–13, 2015.

[5] Ananiadou S. Thompson P. Nawaz R. (2014) Event-based text mining for biology and functional genomics. Brief Funct. Genomics, 3, 213–230 10.1093/bfgp/elu015 PMC4499874 24907365

[6] Keseler I.M. Skrzypek M. Weerasinghe D. (2014) Curation accuracy of model organism databases. Database (Oxford), 2014, bau058. 24923819 10.1093/database/bau058 PMC4207230

[7] Keseler I.M. Mackie A. Peralta-Gil M. (2013) EcoCyc: Fusing model organism databases with systems biology. Nucleic Acids Res., 41(Database issue), D605–D612. 23143106 10.1093/nar/gks1027 PMC3531154

[8] Fang R. Schindelman G. Van Auken K. (2012) Automatic categorization of diverse experimental information in the bioscience literature. BMC Bioinformatics, 13, 16. 22280404 10.1186/1471-2105-13-16 PMC3305665

[9] Van AukenFey K.P. Berardini T. Dodson Z. (2012) Text mining in the biocuration workflow: applications for literature curation at WormBase, dictyBase and TAIR. Database (Oxford), 2012, bas040.[WorldCat] 23160413 10.1093/database/bas040 PMC3500519

[10] Van AukenJaffery K. Chan J.J. Muller H.M., (2009) Semi-automated curation of protein subcellular localization: a text mining-based approach to Gene Ontology (GO) Cellular Component curation. BMC Bioinformatics, 10, 228. 19622167 10.1186/1471-2105-10-228 PMC2719631

